# Tin Nanoparticles Encapsulated Carbon Nanoboxes as High-Performance Anode for Lithium-Ion Batteries

**DOI:** 10.3389/fchem.2018.00533

**Published:** 2018-10-31

**Authors:** Ziming Yang, Hong-Hui Wu, Zhiming Zheng, Yong Cheng, Pei Li, Qiaobao Zhang, Ming-Sheng Wang

**Affiliations:** ^1^Department of Materials Science and Engineering, College of Materials and Pen-Tung Sah Institute of Micro-Nano Science and Technology, Xiamen University, Xiamen, China; ^2^Department of Chemistry, University of Nebraska-Lincoln, Lincoln, NE, United States

**Keywords:** lithium-ion battery, anode material, yolk-shell structure, Sn@C nanoboxes, electrochemical performance

## Abstract

One of the crucial challenges for applying Sn as an anode of lithium-ion batteries (LIBs) is the dramatic volume change during lithiation/delithiation process, which causes a rapid capacity fading and then deteriorated battery performance. To address this issue, herein, we report the design and fabrication of Sn encapsulated carbon nanoboxes (denoted as Sn@C) with yolk@shell architectures. In this design, the carbon shell can facilitate the good transport kinetics whereas the hollow space between Sn and carbon shell can accommodate the volume variation during repeated charge/discharge process. Accordingly, this composite electrode exhibits a high reversible capacity of 675 mAh g^−1^ at a current density of 0.8 A g^−1^ after 500 cycles and preserves as high as 366 mAh g^−1^ at a higher current density of 3 A g^−1^ even after 930 cycles. The enhanced electrochemical performance can be ascribed to the crystal size reduction of Sn cores and the formation of polymeric gel-like layer outside the electrode surface after long-term cycles, resulting in improved capacity and enhanced rate performance.

## Introduction

Over the past few decades, rechargeable secondary lithium-ion batteries (LIBs) have become one of the most popular energy sources for electric vehicles, various portable devices, and grid-scale storage systems because of their high capacity, terrific safety, and steady cycling performance (Bruce et al., 2012; Mahmood et al., 2016; Li et al., 2017b; Liu et al., 2018). However, with the increasing demand of longer usage time and higher capacity about the batteries, graphite, as the widely used commercial anode materials, gradually cannot meet the market needs for its relatively low theoretical capacity of 372 mAh g^−1^ (Hu et al., 2012a; Lim et al., 2012; Leng et al., 2016; Zhang et al., 2018b). Therefore, extensive researches of alloy-based materials (Si, Ge, Sn, etc.) have been studied in LIBs for the higher capacity of these anode materials (Obrovac and Chevrier, 2014; Li et al., 2017a; Wang et al., 2017, 2018; Zhang et al., 2018a). Metallic Sn was reported with high theoretical capacity (997 mAh g^−1^), good electroconductibility and non-toxicity (Hassoun et al., 2007; Hu et al., 2008, 2011, 2012b). However, the giant volume change of 260% during lithiation/delithiation process would result in the pulverization of the active materials after long cycles, and thus the fast capacity fading (Rhodes et al., 2012).

To solve these problems, reducing the size of Sn particles seems to be a good solution because the nano-particles could endure higher stress and effectively prevent the active materials from pulverization (Leng et al., 2015). Nevertheless, nanoparticles prefer to aggregate during the charge and discharge process. A promising strategy to mitigate this drawback is the smart hybridization of Sn with carbon-based materials (graphene, carbon nanotube, etc.) to Sn@carbon composites (Zhang et al., 2014; Huang et al., 2015; Cheng et al., 2016; Qin et al., 2016), wherein the carbon materials could significantly prevent aggregation, buffer the stress concentration resulted from volume expansion and enhance the electroconductibility. Among them, the yolk@shell structure of Sn@C composites is a preferable choice because the hollow space between the core and carbon shell could accommodate more volume variation free from breaking the carbon shell, which could improve the performance of active materials compared with the Sn@C core@shell composites (Zhang et al., 2014, 2017; Qin et al., 2016).

Inspired by the previous work, herein, we synthesize Sn@C nanoboxes with yolk-shell nanostructure comprising a sphere-like Sn core within a hollow cavity surrounded by carbon nanobox via a one-pot spray pyrolysis process followed by hydrogen-thermal reduction. By virtue of structural advantages, the as-prepared electrode exhibits an outstanding reversible capacity of 675 mAh g^−1^ at a current density of 0.8 A g^−1^ after 500 cycles. The crystal size reduction of Sn cores and the formation of polymeric gel-like layer outside the electrode surface during cycling could explain the increase of the reversible capacity during long cycles. The current work shed light on the improvement of anode materials for next-generation high-performance LIBs.

## Experimental

### Synthesis of ZnSnO_3_ nanocubes

The ZnSnO_3_ nanocubes are fabricated via a hydrothermal precipitation method according to the literature (Zhang et al., 2017). In a typical process, 2.875 g (10 mmol) of zinc sulfate heptahydrate (ZnSnO_4_·7H_2_O) is added into 100 mL deionized water and stirred till completely dissolved, and then 20 mL of sodium stannate solution (NaSnO_3_·3H_2_O, 2.667 g, 10 mmol) is added into above zinc sulfate heptahydrate solution. The solution turns into milky immediately and the mixed solution is stirred at 70°C for 4 h. After the reaction, the precipitates are collected by centrifugation and washed with deionized water and alcohol 3 times and dried at 60°C for one night.

### Synthesis of Sn@C nanoboxes

According to the literature (Zhang et al., 2017), 0.5 g ZnSnO_3_ nanoparticles, 0.46 cetyltrimethylammonium bromide, and 14.08 mL H_2_O are added into a beaker before 0.5 h ultrasonic treatment and 1 h stirring, then 0.7 g resorcinol, 56.0 mL absolute ethanol and 0.2 mL NH_3_·H_2_O are added successively and stirred for 0.5 h at 35°C. Finally, 0.1 mL formaldehyde is added by dropwise. After 6 h stirring and polymerization by aging over one night, the obtained ZnSnO_3_@resorcinol formaldehyde (RF) nanoboxes are collected by centrifugation and washed with deionized water and alcohol 3 times respectively. The yolk-shell Sn@C powder is obtained by heated the ZnSnO_3_@RF at 600°C for 5 h with a rate of 2°C min^−1^ under H_2_ (5%)/Ar (95%) atmosphere.

### Synthesis of Sn NPs

1.0 g ZnSnO_3_ nanoparticles are heated at 600°C for 5 h with a rate of 2°C min^−1^ under H_2_ (5%)/Ar (95%) atmosphere.

### Characterizations

The crystal structure is recorded by a powder X-raying diffraction (Ri gaku Ultima IV). The structure and morphology of the samples are investigated via an FEI Talos-F200s transmission electron microscope (TEM) and a Zeiss SUPRA 55 scanning electron microscope (SEM). Thermogravimetric analysis is investigated by an SDT Q600 Simultaneous TGA/DSC instrument with a heating rate of 10°C min^−1^ in the air.

### Electrochemical measurements

The electrochemical measurements are carried out by employing CR2025 coin cells and the working electrode is synthesized via mixing the active materials, conductive acetylene black and sodium carboxymethylcellulose with a weight ratio of 8:1:1. The slurry is coated on a Cu foil and dried at 80°C inside a vacuum oven for 12 h. The Cu foil is first cut into circular disks with a diameter of 14 mm, and the masses of the as-obtained Cu foil circular disk (m_1_) and the slurry coated on the Cu foil circular disk after dried (m_2_) are determined using a microbalance (Mettler Toledo XS3DU) with an accuracy of 1 μg. The active mass of mass loading of the electrode is then calculated as 0.8^*^ (m_2_ – m_1_) and about 0.8 mg cm^−2^. The measured specific capacities of the electrodes are based on the total active mass loading of Sn@C. Li foil is taken as a counter electrode and 1 M LiPF_6_ is mixed with ethyl carbonate, dimethyl carbonate, and diethyl carbonate (EC/DMC/DC = 1:1:1, volume ratio) are used as the electrolyte. The cells are assembled in the Ar-glovebox under Ar atmosphere with both oxygen and moisture below 0.1 ppm. Galvanostatic charge-discharge cycles are tested in the CT2001A LAND battery tester with potential windows of 0.01–3.00 V. And cycle voltammetry measurements are conducted via a CHI660E electrochemical workstation under a scanning rate of 0.1 mV s^−1^. Electrochemical impedance spectroscopy (EIS) is measured in the frequency range from 0.01 to 100 kHz at open circuit potential with an amplitude of 5 mV.

## Results and discussion

**Figure 1:**
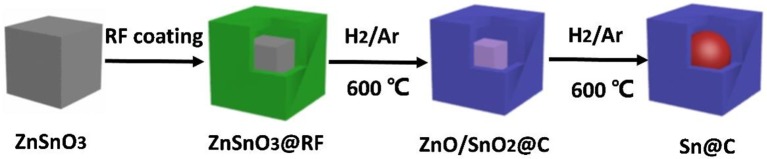
Schematic diagram of the fabrication procedure of yolk-shell Sn@C nanobox.

As illustrated in Figure 1, the resorcinol-formaldehyde (RF) is coated outside of the cubic ZnSnO_3_ nanoboxes, and then the ZnSnO_3_@RF is heated at 600°C under the H_2_ (5%)/Ar atmosphere for 300 min (Zhang et al., 2017). During this process, resorcinol formaldehyde is gradually turned into an amorphous carbon shell while SnO_2_ and ZnO are reduced to metallic Sn and Zn. Due to the low boiling point (907°C), Zn gradually evaporates from the material, which finally generates the yolk-shell Sn@C composite (Zhang et al., 2014). Compared with other reported methods (Zhang et al., 2008; Ni et al., 2013; Wang et al., 2013a, 2015), this one-pot spray pyrolysis method could product yolk-shell Sn@C composite more efficient and controllable.

**Figure 2:**
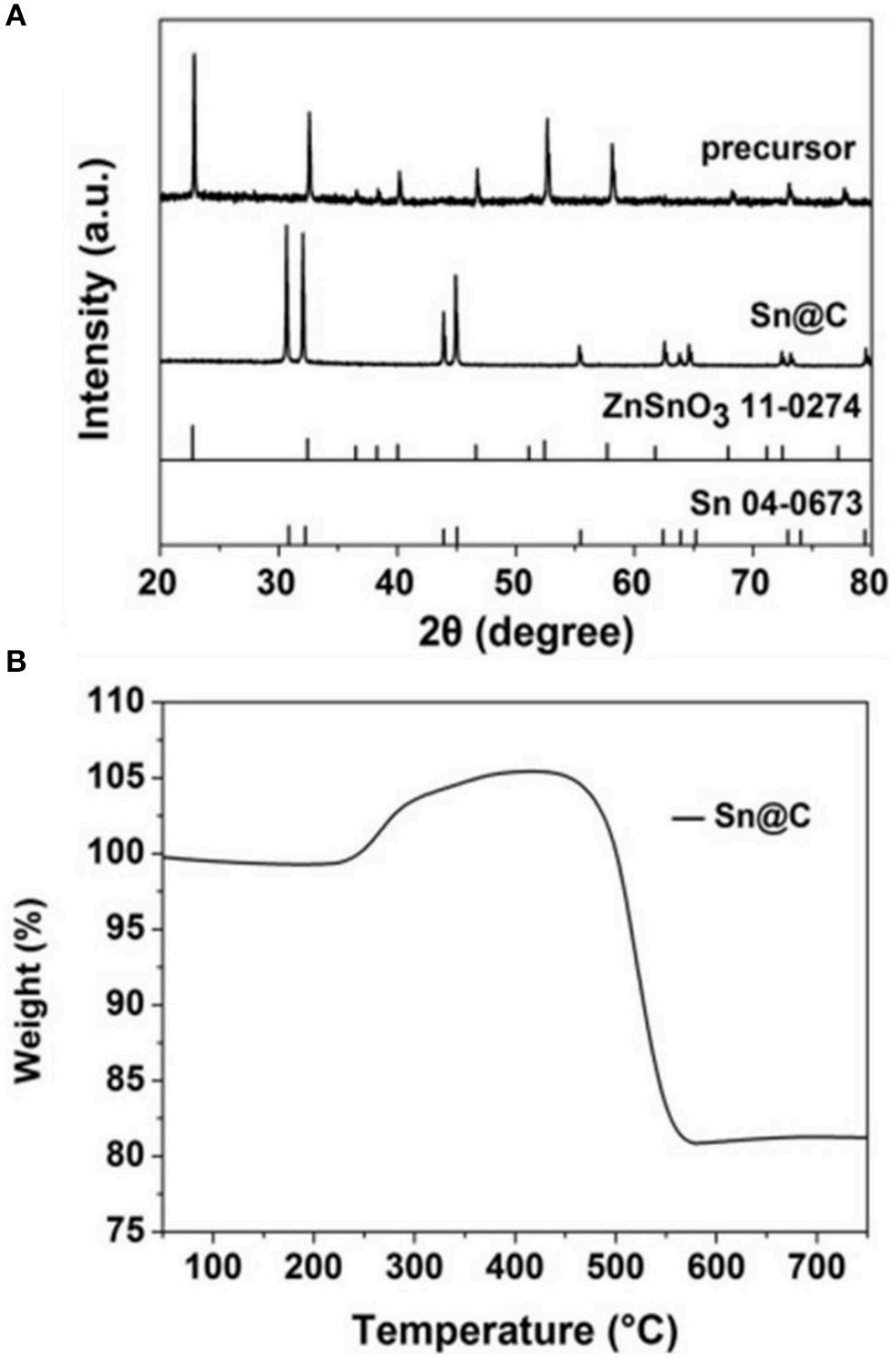
**(A)** XRD pattern of precursor and Sn@C, **(B)** TGA profile of Sn@C.

The X-ray diffraction patterns of the ZnSnO_3_ and Sn@C are shown in Figure 2A. Most peaks of the precursor could be allocated to the ZnSnO_3_ phase (JCPDS NO: 11-0274). After hydrogen-thermal reduction, the final product shows sharp peaks which are indexed to the Sn Phase (JCPDS No: 04-0673). Obviously, there are no obvious diffraction peaks from Zn, SnO_2_, or ZnO, which suggests that most Zn and O had been removed from the precursor. The thermogravimetric curve of the material after hydrogen-thermal reduction (Figure 2B) shows that the loss of weight below 200°C is ascribed to the evaporation the residual moisture. In addition, the distinct loss from 450 to 560°C is attributed to the carbon oxidization whereas the increase from 200 to 450°C is caused by the gradual oxidization of Sn to SnO_2_. The slight increase after 450°C may be attributed the further oxidization of some inner Sn that is not oxidized completely. Assuming the final materials is SnO_2_, the carbon content of the Sn@C nanoboxes is about 21% from the following equation:

$$\begin{array}{l} \text{Sn }(\text{wt}\%)=\text{100 }\times \;\frac{\text{molecular weight of Sn}}{\text{molecular weight of SnO2}}\\ \;\times \frac{\text{final weight of SnO2}}{\text{initial weight of Sn}@\text{C}} \end{array}$$

**Figure 3:**
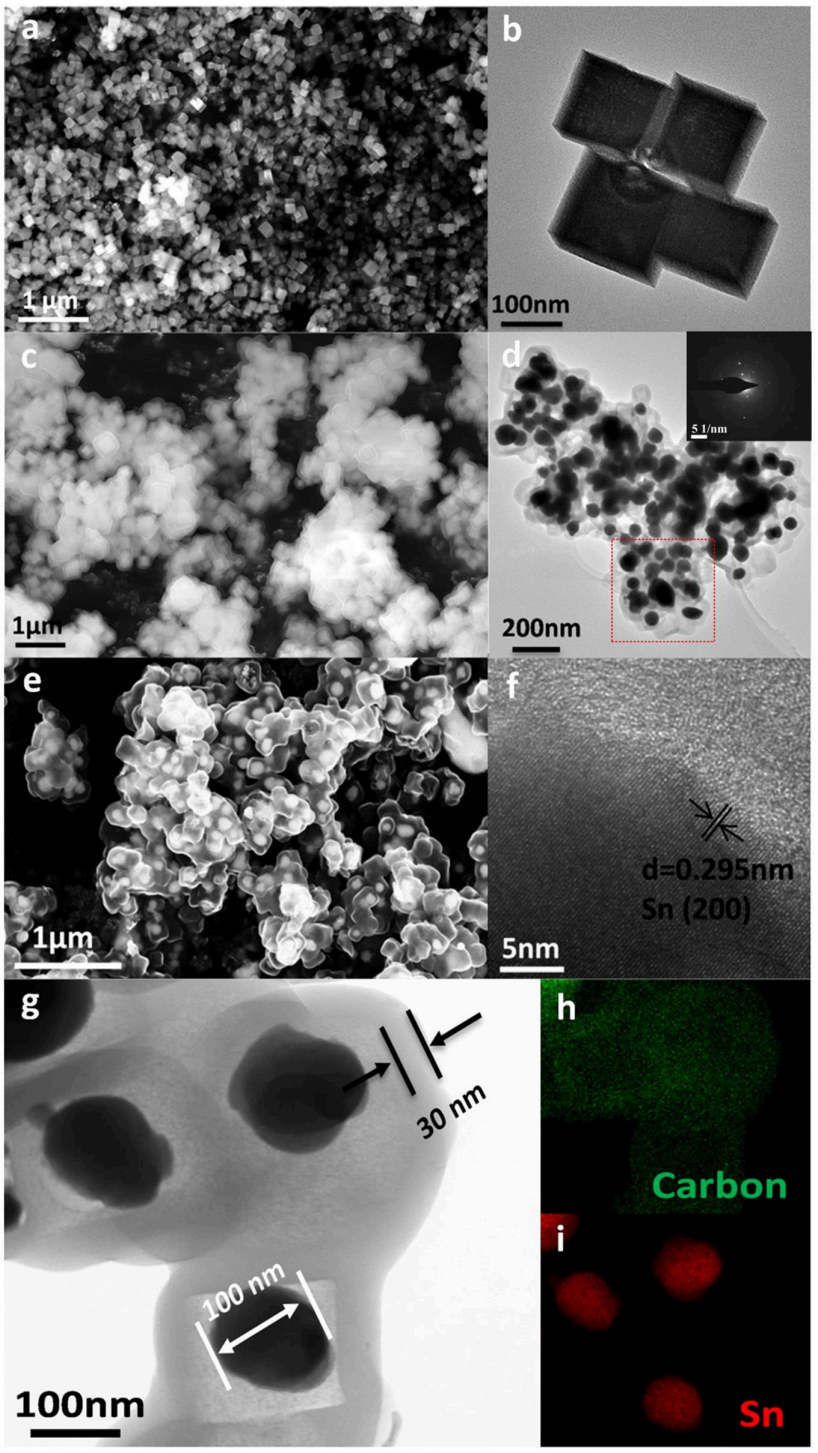
**(a)** SEM, **(b)** TEM image of cubic ZnSnO_3_, **(c)** SEM of ZnSnO_3_@RF, **(d)** TEM, inset is its corresponding SAED patterns taken from the rectangular area, **(e)** SEM, and **(f)** high-resolution TEM image of Sn@C, **(g)** its corresponding STEM image, **(h,i)** corresponding elemental mapping of **(h)** Carbon and **(i)** Sn, respectively.

Scanning electron microscope (SEM) and TEM are employed to observe the morphology and structure of the active materials. As presented in Figures 3a,b, the size of cubic ZnSnO_3_ ranges from 180 to 200 nm. The element mapping and energy dispersive spectrometer of ZnSnO_3_ are shown in Figures S3, S4, respectively. The cubic ZnSnO_3_@RF is fabricated successfully by coating the RF with a uniform thickness of ~20 nm (Figure 3c). After hydrogen-thermal reduction treatment, the outside RF transforms into amorphous carbon while the inside ZnSnO_3_ gradually decomposed, leading to a majority of Zn volatilize from the carbon shell for the relatively low boiling point (907°C). However, Sn is conserved because of the relatively high boiling point (2,260°C). The remaining Sn transforms into the spherical structure to form the yolk-shell Sn@C structure when it is cooled to room temperature. According to Figures 3d,e,g, the TEM and SEM images clearly show the yolk-shell structure where the material in core position is Sn nanoparticles (~100 nm) and the outside shell is carbon (~30 nm). The well-designed yolk-shell structure could completely contain the Sn nanoparticles in each carbon shell and prevent the aggregation of nanoparticles. The selected-area electron diffraction (SAED) patterns shown in the inset of Figure 3d demonstrate the highly crystalline of Sn cores and amorphous nature of carbon shell. A small amount of Sn outflows from the carbon shell, which may be caused by the ultra-small holes in the carbon shell. As shown in Figure 3f, the high-resolution TEM image displays a series of parallel fringes with a space around 0.295 nm, which can be indexed to the (200) plane of crystalline Sn (JCPDS no. 04-0673). Furthermore, the STEM image (Figure 3g) and element mapping image in Figures 3h,i shows that the Sn nanoparticles locate in the carbon shell without any Zn signal, which suggests that most Zn is removed from the shell during the thermal treatment. Besides, the void space in the yolk-shell structure is about 60%, which is designed to accommodate the volume variation during the Li^+^ insertion/de-insertion reaction. As a comparison, the XRD and SEM images of Sn NPs are shown in Figures S1, S2.

**Figure 4:**
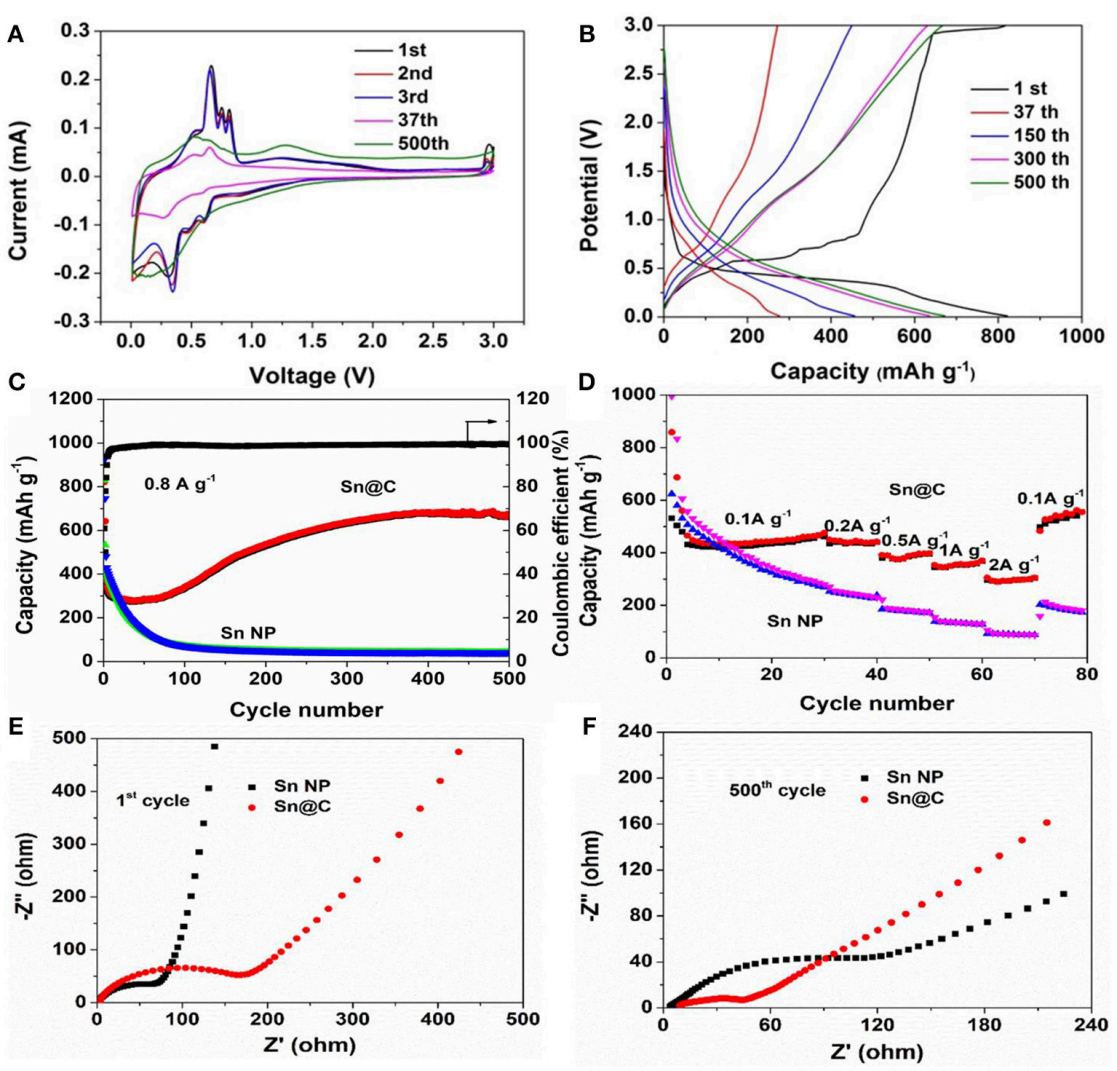
**(A)** CV curves of Sn@C at a scanning rate of 0.1 mV s^−1^ between 0.1 and 3 V, **(B)** charge-discharge curves of Sn@C at 0.8A g^−1^, **(c)** cycling performance of Sn@C and Sn NPs at 0.8 A g^−1^, **(D)** rate capacity of Sn@C and Sn NPs, **(E)** Nyquist plots of samples before cycle, **(F)** Nyquist plots of samples after 500 cycles.

Cyclic voltammograms of the Sn@C electrode (Figure 4A) are tested to understand the electrochemical reactions during the charge/discharge processes. As can be seen, during the first cathodic scan, the three small peaks at 0.25, 0.5, and 0.55 V can be attributed to the alloying reaction between lithium and tin, forming of Li_x_Sn alloys (Dai et al., 2016). During the first anodic scan, four oxidation peaks between 0.40 and 0.80 V are assigned to the delithiation process of Li_x_Sn alloys (Li et al., 2013; Liu J. et al., 2015). Besides, the broad peak at 1.25 V is caused by the Li^+^ extraction from carbon. In addition, there is a peak at 2.85 V in the first anodic scan, which disappears in the following cycles, and may be due to solid electrolyte interface (SEI) layer decomposition induced by nanoscaled Sn particles (Dai et al., 2016). The difference between the first and following cycles are caused by the electrolyte decomposition and formation of SEI films (Luo et al., 2012; Xu et al., 2012; Liu Y. et al., 2015). Figure 4B exhibits the charge and discharge profiles of the yolk-shell Sn@C nanoboxes at a current density of 0.8 A g^−1^ over a voltage range from 0.01 and 3.0 V. Compared with the first cycle of charge curve, the plateau at 0.48 V of the following cycles gradually disappears, besides, the charge and discharge curves become sloping, indicating a linear time-dependent change of potential at a constant current. Combined with the CV curves, the slope curves could be caused by the diffusion-controlled reaction and the capacitive capacity. The disappearance of potential plateaus in the following cycles and the linearization of the charge-discharge curve indicate the extra pseudocapacitive contribution (Augustyn et al., 2013; Li et al., 2015; Xu et al., 2016). The charge and discharge profiles of the Sn NPs at 0.8 A g^−1^ over a voltage between 0.01 and 3.0 V are given in Figure S5. To further explore the electrochemical properties of the active materials, long cycling test (Figure 4C) is carried out. The Sn@C yolk-shell materials deliver initial discharge and charge capacities of 821.5 and 500.3 mAh g^−1^ at 0.8 A g^−1^ in a voltage range from 0.01 to 3 V, corresponding to the first coulombic efficiency of 60.9% (Figure 4C). The largely irreversible capacity loss at the first cycle is related to the formation of SEIs consuming active Li, and can be compensated by prelithiation through either chemical or electrochemical methods or by using stabilized Li metal powder (Zhang et al., 2018a; Zheng et al., 2018a). The reversible capacity quickly decreases to 272.3 mAh g^−1^ at 37th cycle, which could mainly be attributed to the structural degradation and reorganization (Sun et al., 2014). Interestingly, the coulombic efficiency gradually increases to 98.5% from the 2nd cycle to 37th cycle, indicating that a relatively stable SEI layer forms during the initial cycles and renders the active surface substantially inert to further electrolyte decomposition, despite the extreme volume changes experienced by the underlying material during discharge/charge (Zhang et al., 2018a). The capacity after 37th cycle gradually increases to 674.6 mAh g^−1^ after 500 cycles, suggesting an excellent cycling performance. Such an improved performance is mainly due to the following features: the Sn@C yolk-shell structure could steadily encapsulate the Sn nanoparticles and the hollow space between the carbon shells and the Sn cores could effectively buffer expansion caused by the giant volume expansion. Such an increased capacity has also been observed in the other Sn/C composites, SnO_2_/C composites and other metal oxide composites (Wang et al., 2010, 2012, 2013b; Guo et al., 2013; Xu et al., 2013; Liu Y. et al., 2015). It is general for various metal (Cheng et al., 2016) or metal oxides (Sun et al., 2013; Sn, Wang et al., 2013b, Mn, Yu et al., 2006; Liu et al., 2014; Xiao and Cao, 2015; Lian et al., 2017, Co, Laruelle et al., 2002, Fe, Zheng et al., 2018b) electrodes to exhibit such a capacity rise. Recent studies show that the capacity rise may be caused by the reversible formation and decomposition of an organic polymeric/gel-like layer from the electrolyte decomposition. And the polymeric/gel layer outside the active materials could enhance the mechanical cohesion and provide extra Li^+^ insertion sites at the interface of the active materials, which is called “pseudo-capacitance-type behavior” (Wang et al., 2012). Moreover, the gradually increased intensity of the peaks at about 0.6 V (Figure 4B) may be due to the reversible decomposition of the organic polymeric/gel- like layer. It has been confirmed that the layer forms at low voltage and decomposed at high voltage. On the contrary, the Sn NPs shows an initial reversible discharge capacity of 923.3 mAh g^−1^ under the same conditions and then the capacity gradually decreases to 71.4 mAh g^−1^ after 100 cycles. Besides, the cycling performance of Sn@C and Sn NPs at 0.2 A g^−1^ are presented in Figures S6, S7. Figure 4D demonstrates the rate performance of the Sn@C composite. The reversible capacities at the current density of 0.1, 0.2, 0.5, 1, and 2 A g^−1^ are 499.5, 465.8, 420.0, 389.8, and 320.3 mAh g^−1^, respectively, which are much higher than those of the Sn NPs electrode. Besides, when the current density is recovered to 0.1 A g^−1^, a capacity of 509.0 mAh g^−1^ is obtained and continued to increase after high-rate cycles, indicating that the yolk-shell structure could keep the integrity of the Sn nanoparticles and enable them to work normally at different current density. Figures 4E,F displays the impedance spectra of the Sn@C and Sn NPs, respectively. The semicircle in the high frequency is caused by the electrolyte resistance and charge transfer resistance (Liu et al., 2011). Obviously, the semicircle of Sn@C is much bigger than that of Sn NPs at 1st cycle, suggesting that the amorphous carbon shell hinders the Li^+^ transform at first. In the following cycles, the semicircle of Sn@C gradually decreases but that of the Sn NPs increases, and the semicircle of Sn@C is much smaller than that of Sn NPs at 500th cycle, which indicates that the yolk-shell structure could protect the active materials and decrease the impedance in long-term cycles.

**Figure 5:**
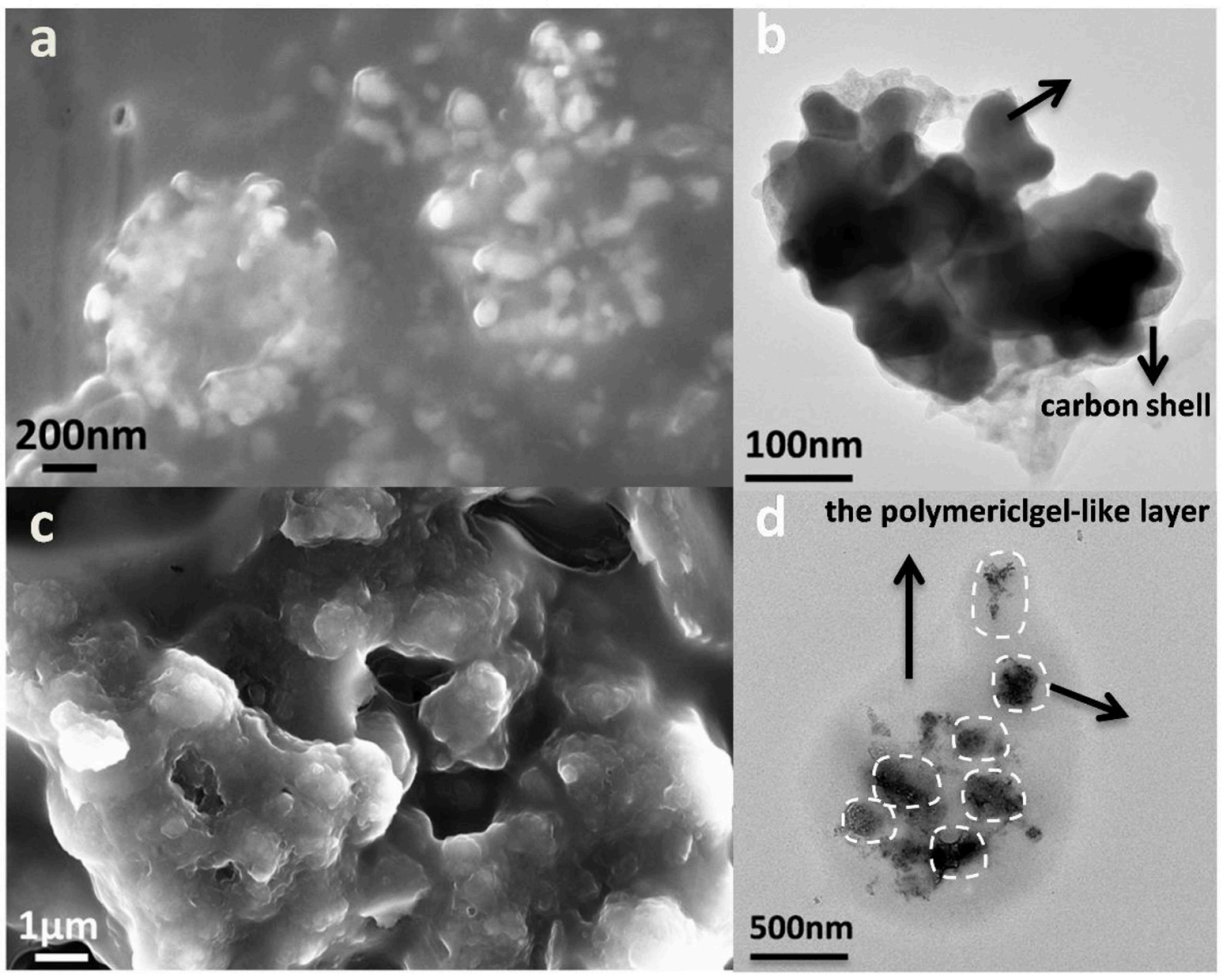
**(a)** SEM and **(b)** TEM of Sn@C after 40 cycles at 0.8 A g^−1^, **(c)** SEM, and **(d)** TEM of Sn@C after 500 cycles at 0.8 A g^−1^.

To further understand the underlying mechanism of the reversible capacity increase for yolk-shell Sn@C composites, TEM and SEM are conducted to capture the Sn@C structure after long cycles at 0.8 A g^−1^. The structure and morphology of Sn@C electrodes after 40 and 500 cycles are observed. As shown in Figures 5a,b, after 40 cycles, most Sn cores swell, which is attributed to the volume change after Li^+^ insertion and extraction process. Additionally, the void space between the carbon shell and Sn core decreases obviously. However, the carbon shell is the same as the pristine state and the Sn nanoparticles are still encapsulated within the shell. Nevertheless, the morphology change is relatively obvious after 500 cycles. According to Figures 5c,d, most of the Sn cores transform into smaller nanoparticles with the diameter range 5–10 nm or even smaller, which is due to the gradually accumulated press/strain caused during the lithiation/delithiation process. Actually, those ultra-small Sn nanoparticles tend to accumulate to a bigger one because the ultra-small nanoparticles have extremely high specific surface area and high surface energy, which could reverse the transform process and cause the rapid capacity decay (Qin et al., 2016). As shown in Figure 5d, a polymericgel-like layer is also formed outside of the Sn@C composite. Such a structure coating outside the Sn@C yolk-shell anode could enhance the mechanical cohesion and offer more lithium interfacial storage sites, which is called “pseudo-capacitance-type behavior” (Purbia and Paria, 2015; Cheng et al., 2016). Moreover, the carbon shell is able to lock the nanoparticle in each carbon nanobox, and the rearrangement of Sn nanoparticles could reduce the stress and strain of the electrode and thus reserve the integrated yolk-shell structure (Xu et al., 2012; Leenheer et al., 2016). Such a phenomenon is observed in MnO (Leenheer et al., 2016), which also exhibits the reversible capacity increase of 800 mAh g^−1^ at 5 A g^−1^ from 200 to 2,000 cycles. Cao etc. explained that more active materials in carbon sheets may crush into small size nanograins upon cycling, which could increase the specific surface to some extent, thus leading to the enhancement of the interfacial Li^+^ storage ability and the improved capacity (Yu et al., 2006; Sun et al., 2013; Liu et al., 2014; Xiao and Cao, 2015; Lian et al., 2017).

**Figure 6:**
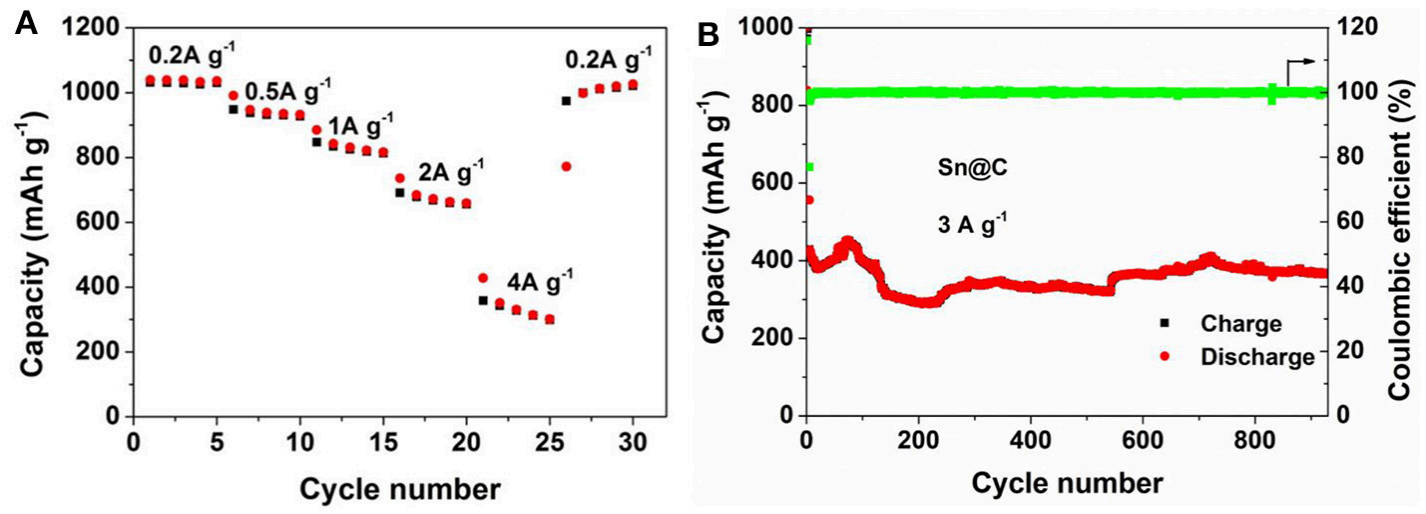
**(A)** Rate capacity and **(B)** cycling performance of Sn@C at 3 A g^−1^ after 500 cycles at 0.8 A g^−1^.

To investigate the improved chemical performance after long cycles, the rate performance, and cycling properties at high current density are measured. As shown in Figure 6A, after 500 cycles at 0.8 A g^−1^, rate performance of Sn@C is tested at 0.2, 0.5, 1.0, 2.0, and 4.0 A g^−1^ and the capacities are 1036.9, 956.0, 839.2, 701.8, 395.8 mAh g^−1^ respectively, which are almost more twice than the capacities of the pristine electrode. From the CV curve (Figure 4A), the electrode also shows relatively smooth curves of charge and discharge after long cycles, which is caused by the combination of capacitive contribution and diffusion-controlled reaction. According to the charge-discharge curves (Figure 4B), the potential plateaus becomes shorter and almost disappears and the curve becomes nearly straight line after long cycles, explaining the increase of the extra pseudocapacity (Purbia and Paria, 2015). Besides, the electrode is also tested at 3 A g^−1^ after 500 cycles at 0.8 A g^−1^, the reversible capacity (Figure 6B) maintains 366.2 mAh g^−1^ after 930 cycles and the coulombic efficient is almost 100%. Compared to previous reports, this yolk-shell Sn@C composite exhibits improved reversible capacity of 694.4 mAh g^−1^ between 16th to 500th cycles at a current density of 0.2 A g^−1^, which have been not observed before (The electrochemical performances of other Sn/C composites are shown in the Table S1). Besides, at a current density of 0.8 A g^−1^, the obvious increase between 37th to 500th cycles approach 402.1 mAh g^−1^. Compared with the Sn NPs without carbon shell, the electrochemical performance of Sn@C is considerably improved. Moreover, this concept can also be employed to other anode materials.

The excellent electrochemical performance is contributed by the following aspects: firstly, the uniform Sn nanoparticles are completely encapsulated in the amorphous carbon shell, which prevents the aggregation of the nanoparticles, and the hollow space between Sn core and carbon shell could significantly accommodate the volume variation to protect the carbon shell from pulverization; secondly, the process of forming ultra-small Sn nanoparticles during lithiation/delithiation to maintain the yolk-shell structure; thirdly, the ultra-small Sn nanoparticles of 10–20 nm after long-term cycles have a larger specific surface area which could provide more Li^+^ insertion location; finally, the polymeric gel-like layer may provide the extrinsic pseudocapacitance after long-term cycles. In conclusion, the transformation of morphology and structure of the yolk-shell Sn@C nanoboxes is mainly responsible for the gradual rise of the reversible capacity and the long-term cycling ability.

## Conclusion

In summary, we report a facile method to synthesize novel Sn@C nanoboxes into yolk-shell structure with enhanced reversible capacity. The as-prepared Sn@C nanoboxes electrode exhibit good electrochemical performance that can respectively deliver a reversible capacity of 674.6 mAh g^−1^ at 0.8 A g^−1^ and 1032.2 mAh g^−1^ at 0.2 A g^−1^ after 500 cycles. There are two main reasons for this distinguished performance: firstly, the Sn nanoparticles are encapsulated completely in the carbon shell and the void space can significantly accommodate the volume expansion. Besides, the SEI film forms outside the carbon shell could maintain steadily during charge/discharge processes. Secondly, the ultra-small Sn nanoparticles gradually crush from the original Sn particles, which can effectively relieve the stress concentration on the electrode during the long cycles. Overall, the proposed concept in this work could be applied to prepare other metal/carbon composites with desired performance in next-generation LIBs electrode materials.

## Author contributions

QZ and M-SW designed this project. ZY carried out the material preparation and electrochemical test; ZY, ZZ, YC, and PL carried out and analyzed the XRD, SEM, and TEM analysis; ZY and H-HW wrote the paper. All authors discussed the results and revised the manuscript.

### Conflict of interest statement

The authors declare that the research was conducted in the absence of any commercial or financial relationships that could be construed as a potential conflict of interest.
