# Supplementary material for: Tin Nanoparticles Encapsulated Carbon Nanoboxes as High-Performance Anode for Lithium-Ion Batteries
Source: Front Chem. 2018 Oct 31;6:533. doi: 10.3389/fchem.2018.00533 (PMC6220033; doi:10.3389/fchem.2018.00533)
Supplement: Supplementary file 1 [file Data_Sheet_1.docx]

Supporting information

**Tin Nanoparticles Encapsulated Carbon Nanoboxes as High-performance Anode for Lithium-Ion Batteries**

Ziming Yang ^a,^ ^#^, Hong-Hui Wu^b,^ ^#^, Zhiming Zheng ^a^, Yong Cheng ^a^, Pei Li ^a^, Qiaobao Zhang ^a, *^, Ming-Sheng Wang ^a,*^

*^a^* Department of Materials Science and Engineering, College of Materials, and Pen-Tung Sah

Institute of Micro-Nano Science and Technology, Xiamen University, Xiamen, Fujian 361005,

China.

*^b^* Department of Chemistry, University of Nebraska-Lincoln, NE 68588 Lincoln, United States

CORRESPONDING AUTHOR

Qiaobao Zhang, E-mail: [zhangqiaobao@xmu.edu.cn](mailto:zhangqiaobao@xmu.edu.cn)

Ming-Sheng Wang, E-mail: mswang@xmu.edu.cn

# These authors contributed equally to this work

The XRD pattern of Sn NPs is exhibited in **Figure S1**, the peak of which could be indexed to the Sn Phase (JCPDS No: 04-0673). As shown in **Figure S2,** the Precursor ZnSnO_3_ is Transformed into irregular Sn NPs(Sn nanoparticles) after heated at 600 °C for 5 h with a rate of 2 °C min^-1^ under H_2_ (5%)/Ar (95%) atmosphere.





**Figure S1.** XRD pattern of Sn NPs


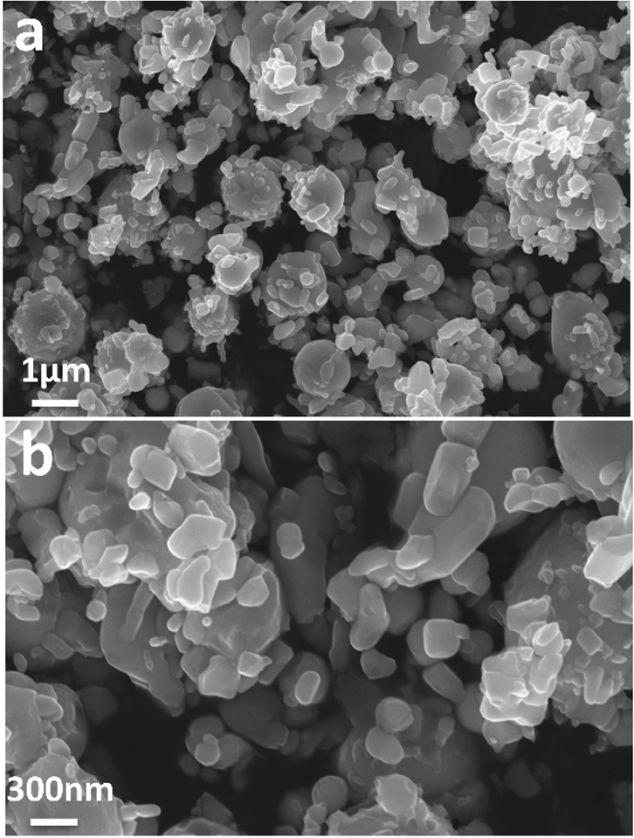


**Figure S2**. SEM images of Sn NP samples.

**The elemental mapping of ZnSnO_3_ is shown in Figure S3 and** the **energy dispersive spectrometer of ZnSnO_3_ is shown in Figure S4.** The peak of Cu is from the copper substrate.


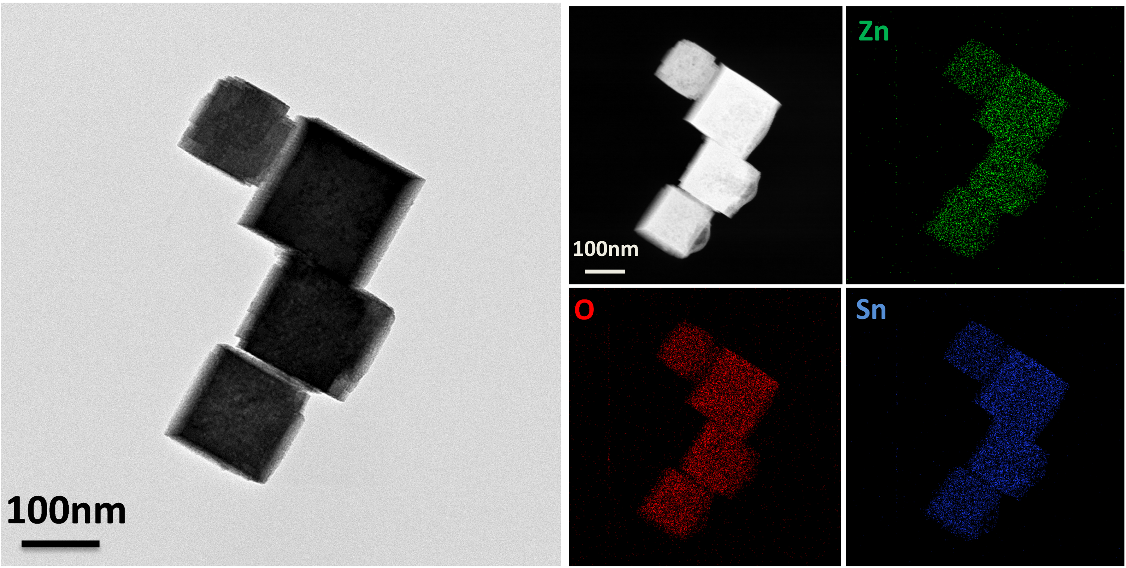


**Figure S3**. **STEM image and its corresponding elemental mapping of ZnSnO_3_.**





**Figure S4. EDX of ZnSnO_3_.**

As shown in **Figure S6 and S7**, it is obvious that the initial reversible discharge capacity of Sn@C is 806.5 mAh g^-1^ at 0.2 A g^-1^ and then the capacity gradually increases to 1032.2 mAh g^-1^ after 500 cycles. However, the Sn NPs shows an initial reversible discharge capacity of 923.3 mAh g^-1^ under the same conditions and then the capacity gradually decreases to 71.4 mAh g^-1^ after 100 cycles.





**Figure S5**. GCD curve of Sn NPs at 0.8 A g^-1^


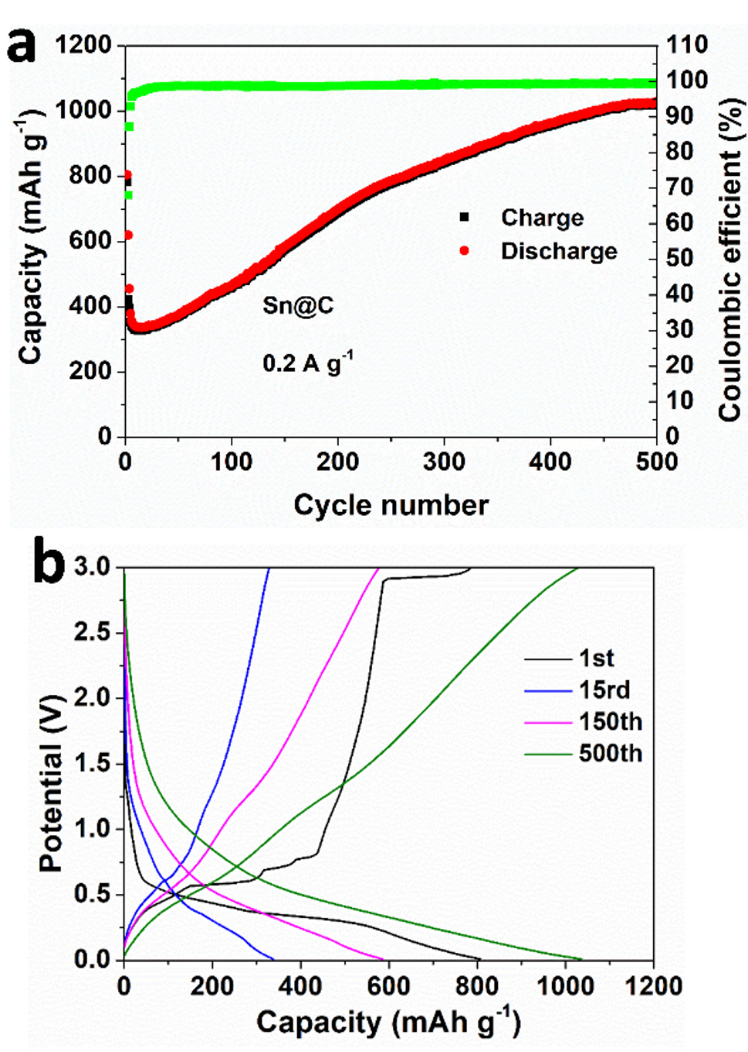


**Figure S6**. (a) cycle performance at 0.2 A g^-1^, (b) the corresponding GCD curve of Sn@C at 0.2 A g^-1^


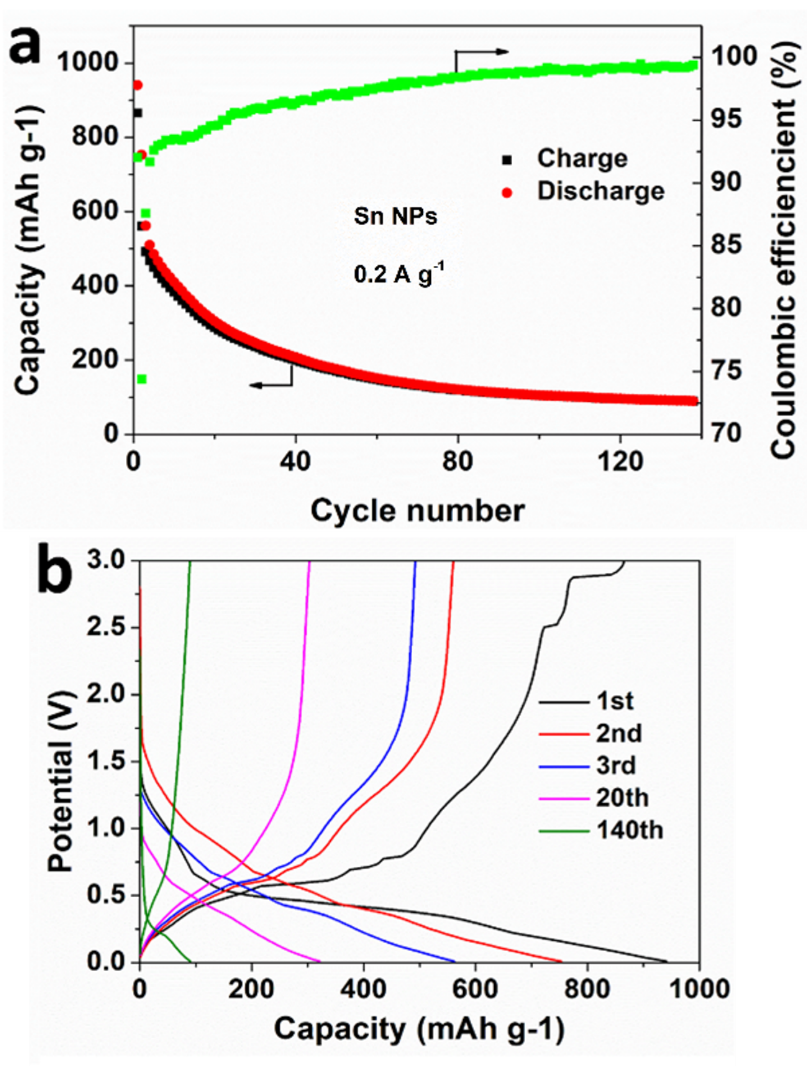


**Figure S7**. (a) cycle performance at 0.2 A g^-1^, (b) its corresponding GCD curve of SnNPs at 0.2 A g^-1^.

| **Table S1** Comparison of electrochemical properties of Sn/C composites for LIBs | | | | |
| --- | --- | --- | --- | --- |
| Sample | Current density  (mA g^-1^) | Cycle numbers | Capacity  (mAh g^-1^) | Reference |
| Sn NP **on CNTs** | 3000 | 1000 | 537 | 7 |
| **Pitaya-like Sn@C** | 200 | 170 | 910 | 8 |
| Sn NP/Carbon | 200 | 200 | **865** | 9 |
| Core-shell Sn seeds | 400 | 600 | 870 | 10 |
| Yolk-shell Sn@C | 200 | 150 | 901 | 11 |
| **Sn@C microsphere** | 1000 | 500 | 537 | 12 |
| **Yolk-shell Sn@C** | 200 | 500 | 810 | 13 |
| Eggette-like Sn@C | 2000 | 800 | 550 | 14 |
| Sn@C nanoshpere | 200 | 800 | 430 | 15 |
| Sn NP/carbon sphere | 200 | 100 | 550 | 16 |
| Core-shell Sn@C | 75 | 100 | 566 | 17 |
| Sponge-like Sn@C | 200 | 300 | 1300 | 19 |
| **Sn@C** | **200**  **800** | **500**  **500** | **1030**  **670** | **This work** |
